# Elevational patterns of soil organic carbon and its fractions in tropical seasonal rainforests in karst peak-cluster depression region

**DOI:** 10.3389/fpls.2024.1424891

**Published:** 2024-10-08

**Authors:** Bei Zhang, Chaohao Xu, Zhonghua Zhang, Cong Hu, Chaofang Zhong, Siyu Chen, Gang Hu

**Affiliations:** ^1^ Key Laboratory of Wildlife Evolution and Conservation in Mountain Ecosystem of Guangxi, Nanning Normal University, Nanning, China; ^2^ Key Laboratory of Ministry of Education for Environment Change and Resources Use in Beibu Gulf, Nanning Normal University, Nanning, China; ^3^ School of Geography and Planning, Nanning Normal University, Nanning, China

**Keywords:** karst, soil organic carbon, recalcitrant organic carbon, labile organic carbon fractions, southwest China

## Abstract

Karst ecosystems, especially tropical karst forests, are crucial to the global carbon cycle. In mountainous and hilly areas, elevation-related changes in environment and vegetation often lead to shifts in the accumulation and decomposition of soil organic carbon (SOC). However, the elevational patterns and influencing variables of SOC and its fractions in tropical karst forest ecosystems remain largely unexplored. Here, we analyzed the elevational patterns of SOC and its fractions in the topsoil and subsoil in the tropical seasonal rainforests within typical peak-cluster depression region of Southwest China. Our results indicated that the SOC content was highest at 400 m asl, which was significantly higher than that at 200 m asl (*p* < 0.05). Overall, SOC content demonstrated an increasing trend with rising elevation. Additionally, SOC content was significantly higher in the topsoil compared to the subsoil (*p* < 0.05). The majority of SOC fractions exhibited an increase with elevation but decrease with soil depth. Notably, only water-soluble organic carbon (WSOC) displayed a decrease with elevation. Meanwhile, recalcitrant organic carbon (ROC, 54.27%), particulate organic carbon (POC, 30.19%), and easily oxidizable organic carbon (EOC, 16.95%) were the main SOC fractions. Labile organic carbon (LOC) in the karst forest soil was predominantly composed of EOC and POC. Correlation analysis unveiled significant positive correlations between SOC and certain fractions with elevation, soil total nitrogen, and exchangeable magnesium. Conversely, significant negative correlations were observed with soil bulk density (SBD), soil total phosphorus, and litter phosphorus (Litter P). Redundancy analysis indicated that elevation, SBD, and Litter P were the main environmental variables influencing shifts in SOC and its fractions. Structural equation models showed that SOC was primarily directly impacted by soil properties but indirectly impacted by elevation. ROC was mainly associated with the direct effects of soil properties and litterfall, although elevation exerted a substantial impact through indirect pathways. Moreover, LOC was predominantly influenced by the direct impact of soil properties. Therefore, this study demonstrates that SOC and its fractions are strongly influenced by elevation in karst peak-cluster depression regions and have important implications for forest management and sustainable ecosystem development in these regions.

## 1 Introduction

The global volume of soil organic carbon (SOC) is approximately 2344 Gt; thus, SOC constitutes the largest terrestrial pool of carbon (Guo et al., 2016a). SOC is derived primarily from organic substances, such as plant and animal residues, root secretions, and microbial metabolites, and plays a pivotal role in mitigating increases in atmospheric CO_2_ concentrations and adjusting the global carbon balance (Ramesh et al., 2019). Factors including forest type, climate, vegetation cover, and soil management also have important roles in the accumulation of forest SOC (Mayer et al., 2020; Wiesmeier et al., 2012). Furthermore, the composition of SOC has different distributions and degradation characteristics in soils at different depths, resulting in significant differences in the SOC content and composition in different types of forests (Guimarães et al., 2013). SOC is divided into labile, slow-acting, and recalcitrant fractions based on its sensitivity to external factors, turnover time, and function (Song et al., 2022). Labile organic carbon (LOC) is a highly reactive form of organic carbon in soil with fast oxidation, decomposition, and mineralization rates, and it can be subdivided into particulate organic carbon (POC), easily oxidizable organic carbon (EOC), water-soluble organic carbon (WSOC), and microbial biomass carbon (MBC) fractions (Li et al., 2018). Although LOC is a small fraction of the SOC, it can reflect minute changes in soil before SOC changes, and it directly participates in biochemical transformation processes. Therefore, LOC more accurately reflects variations in the soil carbon pool than other fractions (Li et al., 2018; Ferraz de Almeida et al., 2019). Compared with labile and slow-acting organic carbon, recalcitrant organic carbon (ROC) is less decomposable and more stable and has a longer turnover time; thus, it is pivotal in maintaining quality and health of forest soil and is frequently employed as an indicator of soil response to environmental shifts (Xu et al., 2010a). To more accurately characterize the dynamics of SOC, different SOC fractions must be investigated.

Elevation is an important geographical factor that significantly impacts ecosystems and climatic conditions. Elevation changes can affect vegetation type, temperature, humidity, soil properties, and climatic conditions and thus can directly or indirectly impact the size and composition of the soil carbon pool (Zhang et al., 2021). Zinn et al. (2018) found that elevation is the dominant variable influencing the SOC content in Brazilian tropical forests. In addition, although increases in elevation correspond to increases in the forest SOC of Mt. Kilimanjaro (Zech et al., 2014) and siliceous Moncayo Massif (Badía et al., 2016), changes in elevation did not result in significant impacts on the SOC in the Peruvian Andes (Zimmermann et al., 2010) and Ecuadorian Andes (Soethe et al., 2007) due to variations in vegetation types and biotic and abiotic factors. The spatial heterogeneity and temporal changes in soil contribute to a high level of uncertainty in estimating SOC at different forest elevations (Mayer et al., 2020). However, few studies have reported on how forest SOC fractions change with elevation. Bojko and Kabala (2017) showed that the content of the most labile carbon fraction decreased with elevation in the Karkonosze Mountains, while Xiang et al. (2015) revealed that the ROC does not change with elevation in the Dinghu Mountains. Thus, analyzing the content and distribution of SOC and its fractions across varying elevations in mountainous and hill areas holds significant importance in elucidating the regional dynamics of the soil carbon cycle.

Karst is a special landform formed by the dissolution and erosion of rock by solifluction. Karst landscapes encompass roughly 22 million km^2^ globally, representing approximately 12% of the Earth’s land area, with a wide and heterogeneous distribution across the globe (Liu et al., 2020). Karst in southwestern China is rich in geological and geomorphological patterns, with unique crested depression landforms created by a combination of conical crests and rounded or polygonal depressions (Guo et al., 2016b). The area is approximately 550,000 km^2^, accounting for nearly 15% of the land area of China (Song et al., 2014). There is a significant heterogeneous spatial distribution of SOC in the karst peak-cluster depressions. Zhang et al. (2008) showed that SOC content is low in depressions and high in peaks with increased elevation. Guo et al. (2017) similarly found that SOC in different habitat types exhibits a pattern of increasing soil carbon content with elevation: peaks > high slopes > mid-slopes ≈ low slopes > depressions. Collectively, these studies show that the SOC content in karst regions tends to increase with elevation; however, the distribution of SOC fractions across varying elevations in karst regions remains poorly understood.

Karst peak-cluster depressions in southwestern China have led to the development of unique and diverse tropical and subtropical karst vegetation ecosystems (Li et al., 2003). Among them, karst seasonal rainforests developed in tropical climates are the most unique forest ecosystems globally and are considered the climax community of soil succession in the cryptic zone (Guo et al., 2016b). Tropical karst seasonal rainforests result from interactions between karst landforms and tropical climates, which have rich biodiversity and important ecological functions. As the mainstay of SOC sequestration in tropical karst areas, tropical seasonal rainforests are critical for soil conservation, the carbon and water cycles, and ecological balance in karst areas. In addition, the region experiences high annual rainfall, is warm and humid throughout the year, and exhibits significant seasonal variation (Bai et al., 2019). Although the soil properties and nutrient cycling (Guo et al., 2017) of tropical karst seasonal rainforests have been investigated, the elevational patterns of SOC and its fractions in tropical forests in peak-cluster depressions remain understudied.

In this study, SOC, ROC, and LOC fractions (including WSOC, EOC, POC, and MBC) content were analyzed in karst seasonal rainforests at four elevation gradients (200, 300, 400, and 500 m). The purposes are to (1) investigate the trends in SOC and its fractions under the four elevational gradients, (2) identify the primary environmental variables affecting the accumulation of SOC and fractions, and (3) determine the pathways through which elevation, soil properties, and litterfall affect SOC and fractions. Our study holds immense significance in enhancing the comprehension of the soil carbon cycle and quantifying the content of carbon and fractions in karst tropical seasonal rainforests across varying elevational gradients.

## 2 Materials and methods

### 2.1 Study area

**Figure 1:**
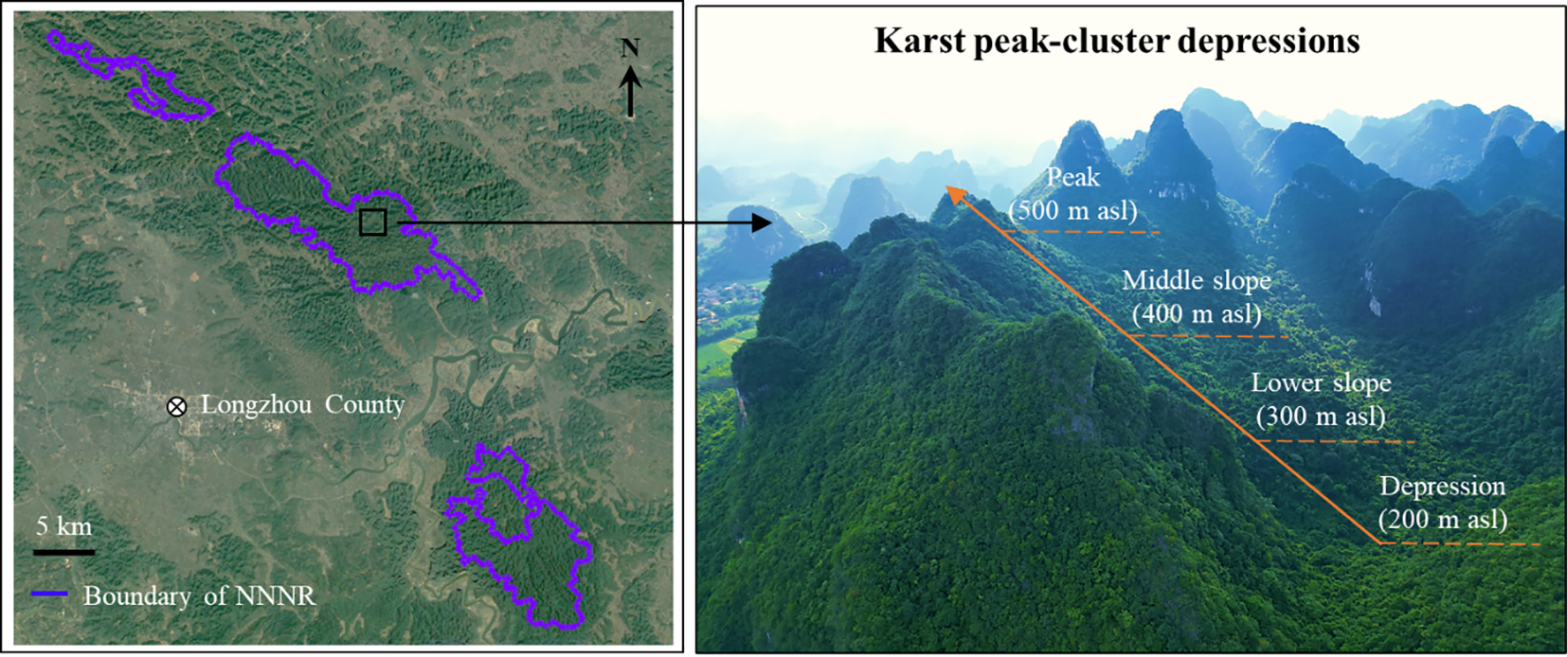
Geographical location of Nonggang National Nature Reserve (NNNR) and its special karst landscape.

The study area was situated in the Nonggang National Nature Reserve (NNNR, E 106°42′28′′–107°04′54′′, N 22°13′56′′–22°33′09′′) in Longzhou County, Guangxi, southwestern China (**Figure 1**). This reserve maintains a typical tropical karst seasonal rainforest with a large area and an intact ecosystem that has not been disturbed by humans for over a century. It is one of China’s 14 key terrestrial biodiversity hotspots of international significance (Guo et al., 2019; Su and Li, 2003); the main protection objectives of the reserve include the limestone seasonal rainforest ecosystem, *Trachypithecus leucocephalus*, *Trachypithecus francoisi*, *Stachyris nonggangensis*, *Parashorea chinensis*, plants of the *Cycas*, and *Theaceae* sect. *Chrysantha* Chang. The reserve has typical karst peaks and depressions that are widely distributed by exposed karst landforms that constitute peaks, forests, depressions, and funnels, forming a variety of regional microclimate types that result in high habitat heterogeneity and compositional complexity in species communities. The reserve features a low elevation ranging from 150 to 600 m, as well as steep slopes. The primary soil type in the reserve is calcareous. The reserve experiences a tropical monsoon climate, primarily influenced by the southeast and southwest monsoons. The area experiences an average annual temperature of 22°C, with an absolute maximum of 39°C and an absolute minimum of –3°C. Annual precipitation ranges from 1150 to 1550 mm, mostly occurring during May to September, with distinct wet and dry seasons. Common tree species include *Excentrodendron tonkinense*, *Cephalomappa sinensis*, *Hydnocarpus hainanensis*, and *Orophea polycarpa*.

### 2.2 Sample collection

In August 2022, four elevations (200 m [depression], 300 m [lower slope], 400 m [middle slope], and 500 m [hill peak]) were selected in karst peak-cluster depressions in the NNNR. At each elevation, three forest plots measuring 20 m × 20 m were established, spaced 20 m apart from each other. The basic information for each site can be found in **Table 1**. Within each plot, a 1 m × 1 m subplot was established using the quincunx method to collect litter for the assessment of litter quantity, as well as the carbon (Litter C), nitrogen (Litter N), and phosphorus (Litter P) content. Moreover, soil samples were collected along with litter from two layers (0–20 cm and 20–40 cm). Five soil samples obtained from the same soil layer within each plot were subsequently combined and divided into two equal portions. One was left to dry naturally and then sieved and used to assess SOC, ROC, WSOC, EOC, POC, and other soil chemical properties, while the other portion was stored at 4 °C for subsequent analysis of MBC. *In situ* soil samples were gathered from each plot using a ring knife (100 cm^3^) to determine the water content (SWC) and bulk density (SBD) (Hossain et al., 2015).

**Table 1:** Basic information for the karst peak-cluster depression region at different elevations.

Elevation (m asl.)	Slope position	Slope (°)	Rock exposure rate (%)	Vegetation cover (%)	Diameter at breast height (cm)	Above-ground biomass (10^3^kg·hm^-2^)	Litter quantity (kg·hm^-2^)	Litter thickness (cm)	Dominant species
200	Depression	5.67 ± 2.52 D	15.00 ± 3.00 D	69.33 ± 4.04 B	6.60 ± 1.47 A	158.79 ± 31.04 A	595.30 ± 41.77 B	2.20 ± 0.20 B	*Litsea variabilis* var. *oblonga*, *Catunaregam spinosa*, *Antidesma bunius*, *Ficus hispida*, *Leea indica*, *Strophioblachia fimbricalyx*
300	Lower slope	19.33 ± 4.04 C	36.67 ± 5.03 C	72.67 ± 2.52 AB	6.66 ± 0.66 A	137.40 ± 17.48 A	679.33 ± 53.87 AB	2.47 ± 0.25 B	*Cephalomappa sinensis*, *Cleistanthus petelotii*, *Hydnocarpus hainanensis*, *Excentrodendron tonkinense*, *Ardisia thyrsiflora*
400	Middle slope	27.67 ± 2.52 B	53.67 ± 3.06 B	76.67 ± 2.89 A	5.33 ± 1.12 AB	122.62 ± 21.62 AB	727.67 ± 45.24 A	3.07 ± 0.25 A	*Hydnocarpus hainanensis*, *Cephalomappa sinensis*, *Orophea polycarpa*, *Pterospermum truncatolobatum*, *Cleistanthus petelotii*, *Cleistanthus sumatranus*
500	Hill Peak	34.00 ± 3.61 A	78.00 ± 4.00 A	57.67 ± 4.04 C	3.89 ± 0.24 B	87.53 ± 11.00 B	491.80 ± 66.22 C	1.70 ± 0.17 C	*Boniodendron minus*, *Cephalomappa sinensis*, *Diospyros siderophylla*, *Lysidice rhodostegia*, *Tirpitzia sinensis*, *Viburnum triplinerve*

Capital letters indicate statistically significant differences among different elevations at *p* < 0.05.

### 2.3 Sample analysis

The SOC was determined using the potassium dichromate oxidation-spectrophotometric method (Chen et al., 2021). Soil ROC was measured by acid hydrolysis (Leavitt et al., 1996). The extraction of soil WSOC followed the procedure outlined by Hao et al. (2022). Soil EOC was determined using the KMnO_4_ oxidation method (Blair et al., 1995). The determination of soil POC followed the protocols outlined by Cambardella and Elliott (1992). Soil MBC was assessed using the chloroform fumigation-extraction method as described by Hao et al. (2022). All soil samples were treated with sulfuric acid prior to organic carbon analysis to remove any potential inorganic carbon, preventing interference from inorganic carbon.

Soil pH was determined using the potentiometric method, while calcium exchange (ECa) was assessed using the ammonium acetate exchange-EDTA complexation titration method, and exchangeable magnesium (EMg) was determined using ammonium acetate exchange-atomic absorption spectrophotometry. Soil total nitrogen (STN) using Kjeldahl nitrogen, soil total phosphorus (STP) using alkali fusion-molybdenum antimony anti-spectrophotometry, and soil total potassium (STK) using atomic absorption spectrophotometry. These soil properties were measured following the methods of Bao (2008). The quantity of litter was determined using a drying method (Morffi-Mestre et al., 2020). The methods of determining C, N, and P contents in litter were the same as those for SOC, STN, and STP, respectively.

### 2.4 Data analysis

Two-way analysis of variance (ANOVA) was performed on SOC and its fractions at different elevations and soil depths using the LSD method (*p* < 0.05) for multiple comparisons. Pearson’s correlation analysis was performed to evaluate the relationships between SOC (and its fractions) and soil physicochemical properties. After removing the multicollinearity among the environmental variables, redundancy analysis (RDA) was implemented to identify the main variables affecting SOC and its fractions and calculate the explanatory rate of environmental variables. The direct and indirect effects of environmental variables on SOC and its fractions were explored by constructing a structural equation model, categorized into three variables to account for two combined variables, including elevation, soil properties (SWC, SBD, and STP), and litterfall (Litter P, Litter N:P, and litter quantity). Two-way ANOVA was conducted in SPSS 24.0, and correlation analysis, RDA analysis, calculation of the explanatory rate of each environmental variable, and structural equation modeling (SEM) were conducted using R packages “psych” (Revelle, 2023), “vegan” (Oksanen et al., 2022), “rdacca.hp” (Lai et al., 2022), and “PiecewiseSEM” (Lefcheck, 2016).

## 3 Results

### 3.1 Elevation patterns of SOC content

**Figure 2:**
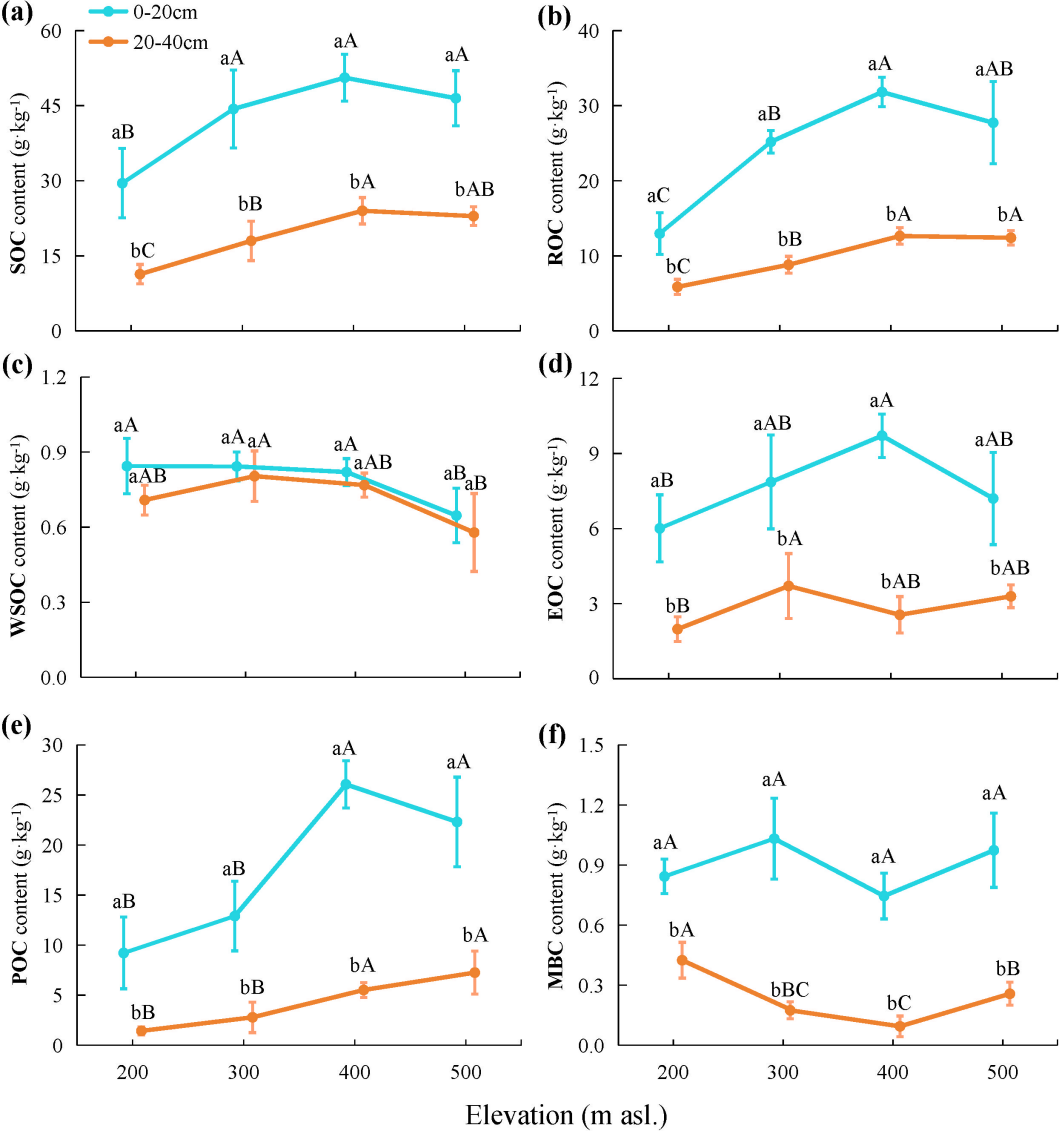
Contents of **(A)** soil organic carbon (SOC), **(B)** recalcitrant organic carbon (ROC), **(C)** water-soluble organic carbon (WSOC), **(D)** easily oxidizable organic carbon (EOC), **(E)** particulate organic carbon (POC), and **(F)** microbial biomass carbon (MBC) in soil collected at different elevations. Lowercase letters indicate statistically significant differences among different soil layers, while uppercase letters denote significant differences among different elevations at p < 0.05.

The results of the two-way ANOVA (**Table 2**) indicated that both elevation and soil depth significantly influenced the SOC content (*p* < 0.001). The SOC content in each soil layer was highest at 400 m asl (**Figure 2A**), which was significantly higher than that at 200 m asl. (*p* < 0.05). Overall, the SOC content showed an increasing trend with higher elevations. The SOC content within the soil profile exhibited a consistent trend across elevation levels, with significantly higher contents detected in the topsoil compared to the subsoil (*p* < 0.05).

**Table 2:** Results of a two-way ANOVA of single and interactions effects of elevation and soil depth on soil organic carbon and its fractions.

Factors	Elevation	Soil depth	Elevation × Soil depth
SOC	13.90***	141.25***	0.96
ROC	31.29***	209.54***	6.71**
WSOC	6.09**	3.68	0.31
EOC	3.47*	91.76***	2.43
POC	21.85***	147.65***	6.61**
MBC	4.28*	188.32***	3.61*
ROC/SOC	5.27*	5.81*	5.35*
WSOC/SOC	10.22**	33.91***	1.83
EOC/SOC	2.24	1.60	1.83
POC/SOC	18.19***	68.44***	2.00
MBC/SOC	24.80***	10.63**	6.18**

F-values listed above are used to assess the magnitude of the effects between two factors. p-values are used to test the significance level of hypothesis testing. *, **, and *** signify *p* < 0.05, *p* <0.01, and *p* < 0.001, respectively. Absence of * indicates *p* > 0.05.

### 3.2 Elevation patterns of SOC fractions

As indicated in **Table 2**, both elevation and soil depth and their interaction had significant impacts on the ROC content (*p* < 0.01). The ROC content in each soil layer was highest at 400 m asl (**Figure 2B**), which was significantly higher than that at 200 and 300 m asl (*p* < 0.05). Overall, the ROC content exhibited an increasing trend with higher elevations. The ROC content in the topsoil at all elevation levels was significantly higher than that in the subsoil (*p* < 0.05). Additionally, the ROC/SOC ranged from 44.15% to 63.02% (**Table 3**), and the two-way ANOVA indicated that neither elevation nor soil depth had a significant impact on the ROC/SOC (*p* > 0.05; **Table 2**).

**Table 3:** Variation in soil organic carbon fractions along varying elevational gradients.

Elevation (m asl.)	Soil depth (cm)	ROC/SOC (%)	WSOC/SOC (%)	EOC/SOC (%)	POC/SOC (%)	MBC/SOC (%)
200	0–20	44.15 ± 1.45 bB	3.04 ± 1.13 bA	20.49 ± 1.84 aA	30.55 ± 5.05 aB	2.93 ± 0.44 aA
	20–40	51.92 ± 3.62 aA	6.42 ± 1.62 aA	17.48 ± 3.21 aAB	12.70 ± 3.09 bB	3.76 ± 0.63 aA
	Average	48.03 ± 4.92 B	4.73 ± 2.24 A	18.99 ± 2.86 A	21.63 ± 10.47 B	3.34 ± 0.67 A
300	0–20	58.16 ± 11.93 aA	1.95 ± 0.43 bAB	17.67 ± 2.54 aAB	28.90 ± 4.33 aB	2.41 ± 0.78 aAB
	20–40	50.41 ± 12.67 aA	4.67 ± 1.38 aAB	20.30 ± 3.15 aA	14.89 ± 4.81 bB	1.00 ± 0.35 bB
	Average	54.28 ± 11.8 AB	3.31 ± 1.75 AB	18.99 ± 2.94 A	21.89 ± 8.70 B	1.71 ± 0.94 B
400	0–20	63.02 ± 2.53 bA	1.64 ± 0.25 bB	19.20 ± 0.64 aA	51.65 ± 4.54 aA	1.48 ± 0.25 aB
	20–40	52.76 ± 1.26 aA	3.24 ± 0.57 aB	10.52 ± 1.92 bC	23.23 ± 4.08 bAB	0.39 ± 0.18 bB
	Average	57.89 ± 5.90 A	2.44 ± 0.96 B	14.86 ± 4.93 A	37.44 ± 16.04 A	0.93 ± 0.63 B
500	0–20	59.24 ± 5.22 aA	1.40 ± 0.23 aB	15.36 ± 2.49 aB	47.64 ± 4.26 aA	2.15 ± 0.69 aAB
	20–40	54.52 ± 8.22 aA	2.54 ± 0.70 aB	14.53 ± 3.20 aBC	31.99 ± 10.97 aA	1.14 ± 0.34 aB
	Average	56.88 ± 6.68 AB	1.97 ± 0.78 B	14.94 ± 2.61 A	39.82 ± 11.35 A	1.64 ± 0.74 B
Average		54.27 ± 8.25	3.11 ± 1.80	16.95 ± 3.84	30.19 ± 14.11	1.91 ± 1.14

Lowercase letters indicate statistically significant differences among different soil layers, while uppercase letters denote significant differences among different elevations at *p* < 0.05.

Two-way ANOVA (**Table 2**) indicated that elevation significantly influenced the content of WSOC, EOC, POC, and MBC. With increasing elevation, the EOC and POC contents tended to increase, WSOC tended to decrease, and MBC exhibited an initially decreasing and then increasing trend, reaching a low value at 400 m asl (**Figures 2C–F**). Additionally, soil depth significantly influenced EOC, POC, and MBC, and the contents were significantly higher in the topsoil at all elevation levels than in the subsoil (*p* < 0.05). Although the WSOC content was higher in the topsoil at all elevation levels than in the subsoil, the difference was not statistically significant (*p* > 0.05). Furthermore, both elevation and soil depth significantly impacted WSOC/SOC, EOC/SOC, POC/SOC, and MBC/SOC. With increasing elevation, WSOC/SOC, EOC/SOC, and MBC/SOC tended to decrease overall while POC/SOC tended to increase. The WSOC/SOC tended to increase with increasing soil depth, while EOC/SOC, POC/SOC, and MBC/SOC tended to decrease.

### 3.3 Correlation between SOC (and its fractions) and environmental variables

**Figure 3:**
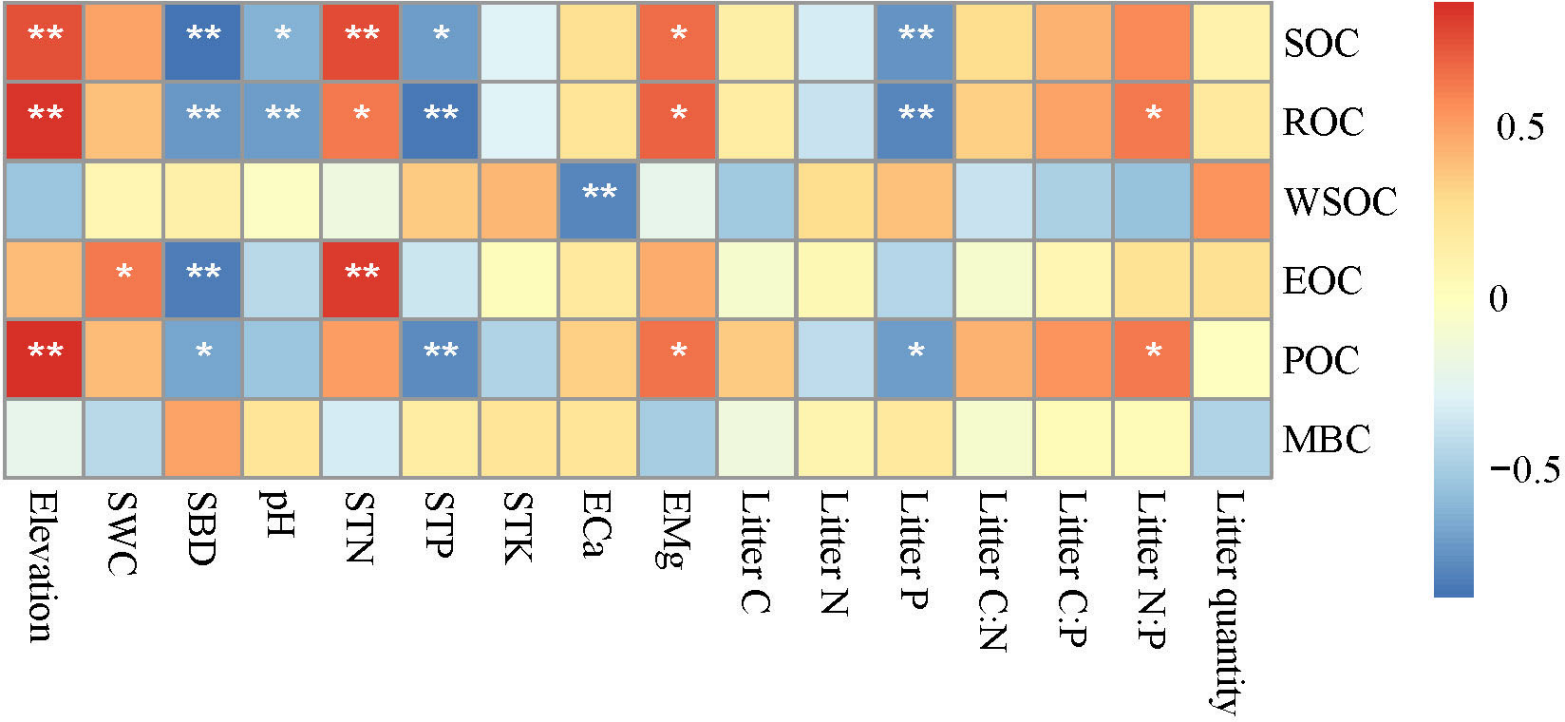
Correlation between soil organic carbon (and its fractions) and soil physical and chemical properties. SOC, soil organic carbon; ROC, recalcitrant organic carbon; WSOC, water-soluble organic carbon; EOC, easily oxidizable organic carbon; POC, particulate organic carbon; MBC, microbial biomass carbon; SWC, soil water content; SBD, soil bulk density; STN, soil total nitrogen; STP, soil total phosphorus; STK, soil total potassium; ECa, exchangeable calcium; EMg, exchangeable magnesium; Litter C, litter organic carbon; Litter N, litter nitrogen; Litter P, litter phosphorus; Litter C:N, litter organic carbon: litter nitrogen; Litter C:P, litter organic carbon: litter phosphorus; Litter N:P, litter nitrogen: litter phosphorus. *p < 0.05, **p < 0.01.

**Figure 4:**
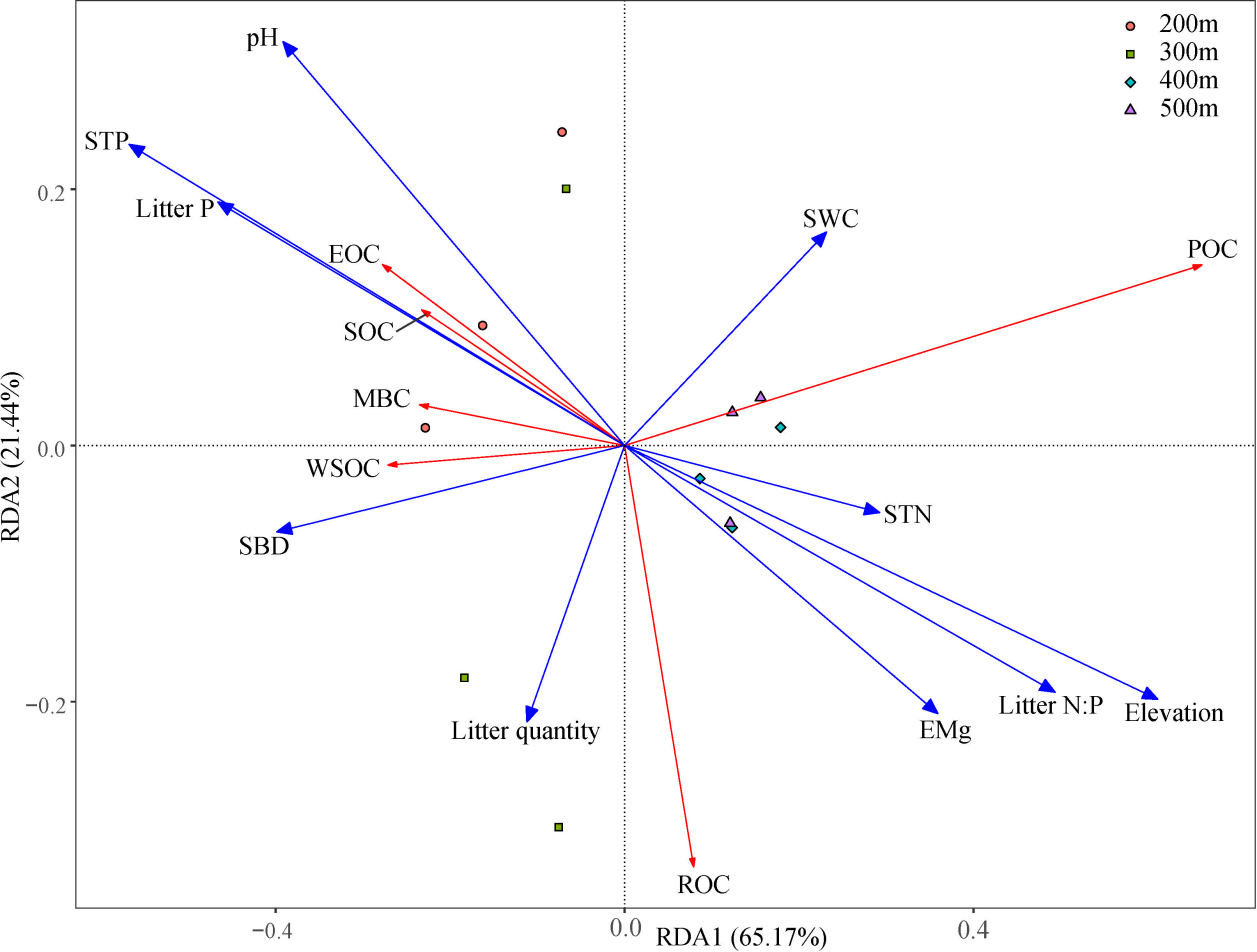
Redundancy analysis of soil organic carbon (and its fractions) with environmental variables. Red lines indicates response variables, and blue lines indicates explanatory variables. SOC, soil organic carbon; ROC, recalcitrant organic carbon; WSOC, water-soluble organic carbon; EOC, easily oxidizable organic carbon; POC, particulate organic carbon; MBC, microbial biomass carbon; SBD, soil bulk density; Litter P, litter phosphorus; SWC, soil water content; STP, soil total phosphorus; STN, soil nitrogen; Litter N:P, litter nitrogen: litter phosphorus; EMg, exchangeable magnesium.

Changes in elevation were accompanied by changes in basic soil physicochemical properties and litterfall properties (**Supplementary Tables A1** and **A2**). Correlation analysis (**Figure 3**) showed that elevation was significantly positively correlated with SOC, ROC, and POC (*p* < 0.01); SWC was significantly positively correlated with EOC (*p* < 0.05); SBD was significantly negatively correlated with SOC, ROC, EOC, and POC (*p* < 0.05 or *p* < 0.01); and pH was significantly negatively correlated with SOC and ROC (*p* < 0.05 or *p* < 0.01). STN and EMg were significantly positively correlated with SOC, ROC, and ROC (*p* < 0.05 or *p* < 0.01); while STP and Litter P were significantly negatively correlated with SOC, ROC, and POC (*p* < 0.05 or *p* < 0.01); and Litter N:P was significantly positively correlated with ROC and POC (*p* < 0.05). The RDA analysis showed that RDA1 and RDA2 accounted for 65.17% and 21.44% of the variance, respectively, and 10 environmental variables explained 96.77% of the variance in SOC and its fractions (**Table 4**; **Figure 4**). Among these, elevation, SBD, and Litter P explained 20.08%, 17.48%, and 11.07% of the variance, respectively.

**Table 4:** Assessment of the explanatory power of individual environmental variables in redundancy analysis.

Environment variables	Volume of explanation (%)	Explanation rate (%)	R^2^	*p*-value
Elevation	20.08	20.77	0.706	0.009
SBD	17.48	18.08	0.689	0.009
Litter P	11.07	11.45	0.557	0.036
SWC	9.87	10.21	0.314	0.184
STP	9.29	9.61	0.547	0.038
STN	8.52	8.81	0.497	0.052
Litter N:P	7.99	8.26	0.522	0.046
EMg	5.18	5.36	0.461	0.067
pH	4.97	5.14	0.346	0.145
Litter quantity	2.29	2.37	0.421	0.102
Total	96.74	100.00	–	–

SBD, soil bulk density; Litter P, litter phosphorus; SWC, soil water content; STP, soil total phosphorus; STN, soil total nitrogen; Litter N:P, litter nitrogen: litter phosphorus; EMg, exchangeable magnesium.

### 3.4 Environmental variable pathways influencing SOC and its fractions

**Figure 5:**
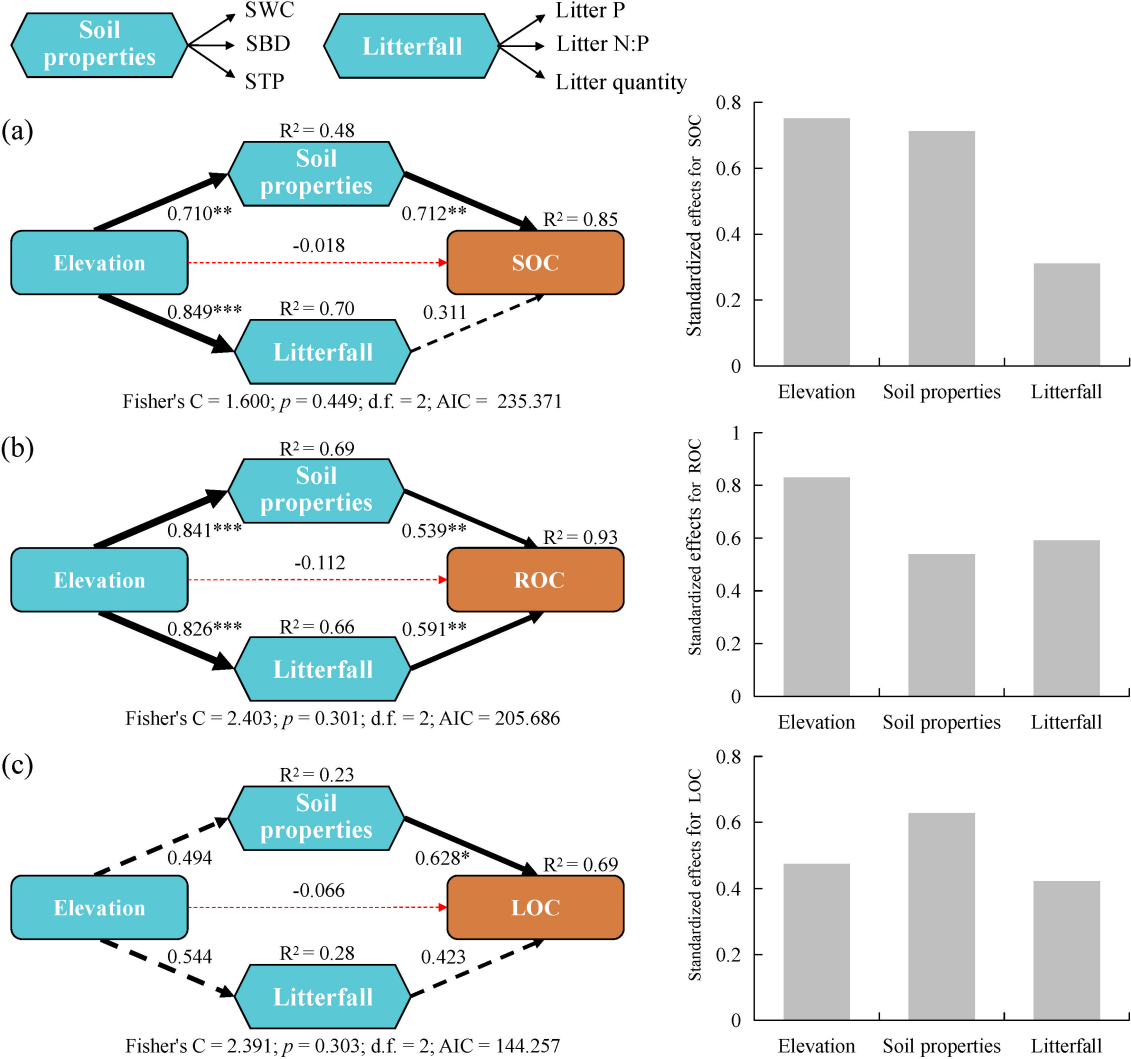
Piecewise SEM of the pathways influencing the accumulation of **(A)** soil organic carbon (SOC), **(B)** recalcitrant organic carbon (ROC), and **(C)** labile organic carbon (LOC) and their standardized effects. LOC was derived from the principal component analysis of four active organic carbon fractions (WSOC, EOC, POC, and MBC), including the first and second principal components. Triangles represents the observed variables, and hexagons indicate composite variables (e.g., soil properties and litterfall). The solid and dashed lines indicate regression results that are significant and not significant, respectively; and red and black lines represent negative and positive pathways, respectively. Numbers next to the arrows represent regression coefficients. The thickness of the line indicates the size of the path coefficient. *p < 0.05, **p < 0.01, ***p < 0.001.

Piecewise SEM analysis (**Figure 5**) showed that elevation soil properties, and litterfall accounted for most of the variations in SOC (85%), ROC (93%), and LOC (69%). SOC was primarily influenced directly by soil properties, with a path coefficient of 0.712 (*p* < 0.01). Additionally, elevation had a significant indirect impact, with standardized effects for SOC reaching 0.752. ROC was mainly influenced directly by both soil properties and litterfall factors, with path coefficients of 0.539 (*p* < 0.01) and 0.591 (*p* < 0.01), respectively. Furthermore, elevation had a substantial indirect impact, with standardized effects for ROC reaching 0.829. LOC was primarily influenced directly by soil properties, with a path coefficient of 0.628 (*p* < 0.05).

## 4 Discussion

### 4.1 Elevational patterns of SOC and its fractions in karst peak-cluster depressions

Changes in elevation are accompanied by variations in slope position and slope degree. Previous studies have suggested that in karst areas, the SOC content in depressions is often lower than that on slopes (Bai and Zhou, 2020; Li et al., 2022), which has been corroborated in this study. This distribution pattern is attributed to the fact that the formation of “rock crevice soil” and “rock bowl soil” due to karst processes leads to the extensive development of stone crevices and stone troughs on slopes. These soil layers are thick and nutrient-rich, and they possess strong water retention capabilities, thus creating favorable conditions for SOC accumulation. In contrast, depressions undergo surface runoff during the formation of standing water despite representing accumulation areas. This results in the preferential erosion of soil aggregates with higher sand content in depressions, thereby hindering the accumulation of SOC in depressions (Zhang et al., 2008). Additionally, a notable phenomenon in karst regions is that the higher the degree of exposed rock, the higher the corresponding SOC content, which aligns with the findings of this study. This might be because a higher rate of rock exposure leads to local accumulation of soil in depressions. In such environments, the accumulation of organic matter and litter is most abundant in micro-landforms such as stone basins, stone troughs, and rock fissures, contributing to higher SOC content (Wang et al., 2020). The SOC content in the topsoil at various elevation gradients was significantly higher than that in the subsoil, which is consistent with the results reported by Bojko and Kabala (2017). The variations in SOC between soil layers may have been caused by the decomposition of plant litter and accumulation of humus and litter in surface soil (Chen et al., 2023). Additionally, with increasing soil depth, the soil texture becomes more compact, limiting the downward transport of surface soil organic matter and resulting in a decrease in accumulated SOC in the subsoil (Chen et al., 2016; Danardono et al., 2019). Furthermore, recent studies have indicated that microclimate variables, particularly soil temperature and light effectiveness, can significantly influence the SOC cycle in karst ecosystems (Huang et al., 2024; Zhao et al., 2024). Future studies should investigate how these variables affect SOC and its fractions in the tropical seasonal rainforests within karst peak-cluster depression region.

The ROC content in our study tended to increase with elevation because with increasing elevation in karst peak-cluster depressions, the terrain steepened, resulting in stronger soil erosion and detachment. Soil erosion carries away lighter soil particles, leaving behind heavier organic matter and higher levels of ROC at higher elevations (Parker et al., 1990). Additionally, the ROC/SOC ratio in this study also tended to increase with elevation, further suggesting that higher elevations improve the stability of SOC to a certain extent. This finding aligns with the study by Okello et al. (2023). In addition, we observed that the ROC content in the topsoil was higher than that in the subsoil. This finding is attributed to two factors: first, the topsoil typically receives a greater amount of plant residues and roots, which contribute to the organic carbon content in the soil and lead to the formation of ROC in the topsoil; and second, topsoil generally exhibits higher biological activity, resulting in increased decomposition and transformation rates of easily decomposable organic matter. As a consequence, the content of ROC is more stable in the topsoil but relatively low in the subsoil due to its limited biological activity (Xiang et al., 2015).

LOC demonstrates rapid responsiveness to soil disturbances and acts as a sensitive indicator for detecting early alterations in SOC levels (Wang et al., 2024). In our study, EOC (16.95%) and POC (30.19%) in the LOC fraction accounted for a significant proportion of SOC (**Table 3**). Consequently, the LOC in the karst forest soil was predominantly composed of EOC and POC. EOC and POC are more effective indicators of shifts in the soil’s labile carbon pool. We observed that both EOC and POC tended to increase with rising elevation, consistent with the observations of Xu et al. (2010b). EOC can be rapidly utilized by microorganisms and other organisms, and the warmer wetter environment at lower elevations promotes increased microbial activity, which accelerates the breakdown and transformation of EOC (Gross and Harrison, 2019). As for the POC content, it was significantly higher in the hill peak and middle slope areas in contrast to the lower slope and depression areas. This is attributed to the pronounced transport and deposition of particulate matter during waterlogging, which may have increased the removal of POC by runoff, resulting in a low POC content in lower slope and depression areas (Zhang et al., 2008). This study also found that the WSOC content tended to decrease with elevation, which may have been linked to the lower vegetation cover at high-elevation sites, relatively lower inputs of plant residues and organic matter, and higher rock exposure, resulting in relatively lower soil microbial activity and reduced release and transformation of WSOC (Huang et al., 2013). In this study, MBC in the subsoil exhibited a trend of initial decline followed by an increase with increasing elevation. This trend corresponds to changes in the pH of the subsoil, suggesting a potential link between elevated pH in the lower soil layer and the ecological environment and activity of microorganisms (Babur et al., 2022). In contrast, topsoil may be more influenced by external factors, such as the input of plant residues and climatic fluctuations, resulting in no apparent trend in MBC in topsoil (Zheng et al., 2022). In the soil profile, we found that the content of EOC, POC, and MBC in the topsoil was significantly higher than in the subsoil. Although WSOC was also higher in the topsoil, this difference was not statistically significant. This trend is closely related to the sources of organic carbon in the topsoil. Higher EOC in the topsoil is due to warmer and more humid conditions that favor microbial activity and organic matter oxidation (Ramesh et al., 2019). POC and MBC levels are higher in the topsoil due to the presence of plant litter, which adds organic matter and supports microbial growth (Prescott and Vesterdal, 2021). The topsoil, being closer to plant roots, benefits from greater organic matter input and better aeration (Philippot et al., 2024). The lack of significant differences in WSOC between soil layers is likely due to its rapid movement and degradation, leading to minimal variation across soil layers (Sanderman and Amundson, 2008).

### 4.2 Impact of environmental variables on SOC and fractions in karst peak-cluster depressions

This study revealed a significant positive correlation between SOC and certain fractions with elevation, STN, and EMg. Conversely, a significant negative correlation was observed between SOC and certain fractions with SBD, STP, and Litter P. On the one hand, higher SOC content is often associated with more organic matter, which potentially provides microbes with additional carbon sources, thereby promoting the cycling and supply of nitrogen and magnesium in the soil (Hu et al., 2020). On the other hand, high SBD typically indicates a compact soil structure, which may restrict the decomposition of organic carbon and the activity of microorganisms, thereby reducing the SOC content (Wang et al., 2021). The negative correlation between SOC and STP and Litter P may reflect a competitive relationship between SOC and phosphorus. Specifically, the increase in organic carbon may stimulate microbial activity, and microbes that decompose organic matter may utilize phosphorus in the soil, resulting in a decrease in phosphorus content (Wang and Sheng, 2023). In addition, elevation, SBD, and Litter P individually explained 20.77%, 18.08%, and 11.45% of the variation in SOC and its fractions, respectively, indicating that these were the main environmental variables responsible for the variability of SOC and its fractions. In this study, elevation had a significant impact on SOC and its fractions. First, increases in elevation lead to changes in topography and climate, thereby influencing vegetation types and growth conditions (Zhong et al., 2022). Second, soil characteristics such as SBD, pH, and nutrient content vary with changes in elevation, thereby directly affecting the stability and decomposition rate of SOC (Zhang et al., 2021). Additionally, elevation may also influence the quality and quantity of vegetation residues and activity level of soil microorganisms, thereby further impacting the accumulation and decomposition of SOC (Babur et al., 2021). Wang et al. (2021) suggested that SBD is the primary negative factor influencing SOC, which aligns with the findings of the current study. In addition, in tropical karst regions, soil phosphorus limitations are commonly observed, which restrict the growth and metabolism of plants and microorganisms. Consequently, SOC accumulates because it cannot be fully utilized (Geekiyanage et al., 2019). Furthermore, the accumulation of soil organic matter leads to increased fixation and adsorption of phosphorus in the soil, which reduces the available phosphorus content in plants and contributes to the negative correlation between STP and SOC, as well as certain organic carbon fractions (Leytem and Mikkelsen, 2005). Litter P enters the soil via decomposition and dissolution and participates in the phosphorus cycle within the soil (Sohrt et al., 2017). However, due to the adsorption and fixation effects of phosphorus, a portion of the phosphorus is unable to migrate effectively into the soil, resulting in decreased effectiveness. This was further reflected in the limited nature of phosphorus in the tropical karst soils.

The distribution of SOC along the elevational gradients was influenced by multiple factors. As elevation increases, climate and environmental conditions change, which affect vegetation growth and soil physicochemical properties. Moreover, variations in plant growth rates and nutrient uptake capacities at different elevations can affect the C, N, and P contents of litter (Körner, 2007). In this study, the content of SOC was primarily influenced by the direct impact of soil properties, while elevation significantly affected SOC content through indirect pathways. ROC was mainly influenced by the direct effects of soil properties and litter factors, with elevation also exerting a substantial impact through indirect pathways. As for LOC, its content was predominantly influenced by the direct impact of soil properties. This suggests that when studying SOC and its fractions, the direct effects of soil properties and indirect impacts of factors such as elevation through complex pathways must be considered. Such factors must be considered to develop a comprehensive understanding of the mechanisms underlying the formation and distribution of SOC. Additionally, changes in elevation can lead to alterations in vegetation structure, such as species diversity, tree density, basal area, and forest floor cover (Pöpperl and Seidl, 2021). Future research should emphasize the inclusion of vegetation metrics to better understand the intricate relationships between vegetation characteristics and SOC distribution in karst environments. This approach will lead to a more comprehensive understanding of the mechanisms driving SOC formation and distribution across different elevations.

## 5 Conclusions

To the best of our knowledge, this is the first study to report the elevational patterns of SOC and its fractions in a tropical seasonal rainforest in the karst peak-cluster depression region of southwestern China. We found that the SOC content was highest at 400 m asl, which was significantly higher than that at 200 m asl. Overall, SOC content demonstrated an increasing trend with rising elevation. Additionally, SOC content was significantly higher in the topsoil compared to the subsoil. The ROC, EOC, and POC content tended to increase while the WSOC content tended to decrease with increasing elevation. SOC fractions (excluding WSOC) were significantly higher in the topsoil than the subsoil. The proportions of the different SOC fractions responded differently to changes in elevation and soil depth, with ROC (54.27%), POC (30.19%), and EOC (16.95%) acting as the main SOC fractions. As the primary LOC fractions, POC and EOC effectively reflected the activity of this soil carbon pool. SOC and its fractions were closely related to various environmental variables at different elevations. Notably, SOC and certain fractions were significantly positively correlated with factors such as elevation, STN, and EMg but significantly negatively correlated with factors such as SBD, STP, and Litter P. Elevation, SBD, and Litter P contributed to variations in SOC and its fractions. SOC was primarily influenced by the direct impact of soil properties and significantly affected through the indirect effect of elevation. ROC was mainly influenced by the direct effects of soil properties and litter factors but also substantially impacted by the indirect effects of elevation. In addition, LOC was predominantly influenced by the direct impact of soil properties. This suggests that when studying SOC and its fractions, it is crucial to consider the direct effects of soil properties as well as the indirect impacts of factors such as elevation through complex pathways.

## Data Availability

The raw data supporting the conclusions of this article will be made available by the authors, without undue reservation.
