# Supplementary material for: Elevational patterns of soil organic carbon and its fractions in tropical seasonal rainforests in karst peak-cluster depression region
Source: Front Plant Sci. 2024 Oct 8;15:1424891. doi: 10.3389/fpls.2024.1424891 (PMC11493711; doi:10.3389/fpls.2024.1424891)
Supplement: Supplementary file 1 [file Table1.docx]

Supplementary Material

Elevational patterns of soil organic carbon and its fractions in tropical seasonal rainforests in karst peak-cluster depression region

Bei Zhang^1,2#^, Chaohao Xu^1#^, Zhonghua Zhang^1,2^, Cong Hu^1^, Chaofang Zhong^1^, Siyu Chen^1^, Gang Hu ^1,2*^

^#^ There authors contributed equally to this work.

*** Correspondence:** Gang Hu: [ahhugang@126.com](mailto:ahhugang@126.com)

# Supplementary Tables

**Table A1** Basic soil physicochemical properties at different elevations.

| Elevation (m asl.) | Soil depth (cm) | SWC | SBD | pH | STN | STP | STK | ECa | EMg |
| --- | --- | --- | --- | --- | --- | --- | --- | --- | --- |
| 200 | 0–20 | 42.11 ± 6.08 aAB | 1.22 ± 0.11 aA | 7.54 ± 0.22 aA | 3.17 ± 0.78 aA | 2.29 ± 0.49 aA | 4.94 ± 0.88 aA | 24.57 ± 0.43 aB | 1.16 ± 0.11 aA |
|  | 20–40 | 33.56 ± 3.83 aA | 1.43 ± 0.09 aA | 7.64 ± 0.30 aA | 1.51 ± 0.22 bB | 2.03 ± 0.30 aA | 4.20 ± 0.69 aAB | 20.58 ± 3.82 aA | 0.94 ± 0.13 aA |
|  | Average | 37.83 ± 6.52 A | 1.33 ± 0.15 A | 7.59 ± 0.24 A | 2.34 ± 1.04 B | 2.16 ± 0.39 A | 4.57 ± 0.82 A | 22.58 ± 3.27 B | 1.05 ± 0.16 B |
| 300 | 0–20 | 44.02 ± 4.97 aAB | 1.10 ± 0.13 aA | 7.19 ± 0.10 aB | 4.56 ± 0.48 aA | 1.29 ± 0.34 aAB | 4.58 ± 1.78 aA | 26.12 ± 2.25 aB | 1.19 ± 0.02 aA |
|  | 20–40 | 34.09 ± 1.81 bA | 1.31 ± 0.08 aAB | 7.13 ± 0.08 aB | 2.92 ± 0.52 bA | 1.07 ± 0.41 aAB | 4.70 ± 1.91 aA | 23.11 ± 1.50 aA | 1.13 ± 0.04 aAB |
|  | Average | 39.06 ± 6.39 A | 1.21 ± 0.15 A | 7.16 ± 0.08 B | 3.74 ± 1.00 A | 1.18 ± 0.36 B | 4.64 ± 1.65 A | 24.62 ± 2.38 B | 1.16 ± 0.04 AB |
| 400 | 0–20 | 49.53 ± 3.46 aA | 1.07 ± 0.08 bA | 7.00 ± 0.03 aB | 4.54 ± 1.26 aA | 0.91 ± 0.21 aBC | 3.81 ± 2.14 aA | 23.49 ± 1.39 aB | 1.25 ± 0.09 aA |
|  | 20–40 | 37.05 ± 4.26 bA | 1.28 ± 0.03 aB | 7.18 ± 0.18 aB | 2.47 ± 0.54 aA | 0.73 ± 0.30 aBC | 3.56 ± 2.64 aAB | 20.69 ± 1.62 aA | 1.17 ± 0.11 aB |
|  | Average | 43.29 ± 7.67 A | 1.17 ± 0.13 A | 7.09 ± 0.15 B | 3.51 ± 1.43 AB | 0.82 ± 0.25 C | 3.69 ± 2.15 A | 22.09 ± 2.04 B | 1.21 ± 0.10 A |
| 500 | 0–20 | 38.68 ± 4.54 aB | 1.12 ± 0.07 aA | 7.06 ± 0.19 aB | 3.71 ± 0.65 aA | 0.42 ± 0.09 aC | 0.90 ± 0.80 aB | 34.43 ± 6.14 aA | 1.26 ± 0.11 aA |
|  | 20–40 | 34.33 ± 0.55 aA | 1.28 ± 0.10 aB | 7.33 ± 0.22 aAB | 2.63 ± 0.21 aA | 0.31 ± 0.05 aC | 1.17 ± 0.76 aB | 29.13 ± 13.08 aA | 1.16 ± 0.10 aB |
|  | Average | 36.50 ± 3.74 A | 1.20 ± 0.12 A | 7.20 ± 0.24 B | 3.17 ± 0.73 AB | 0.37 ± 0.09 D | 1.04 ± 0.72 B | 31.78 ± 9.59 A | 1.21 ± 0.11 A |
| Average | | 39.17 ± 6.38 | 1.23 ± 0.14 | 7.26 ± 0.27 | 3.19 ± 1.14 | 1.13 ± 0.73 | 3.48 ± 2.02 | 25.27 ± 6.34 | 1.16 ± 0.12 |
| Two-way ANOVA | |  |  |  |  |  |  |  |  |
| Elevation | | 3.170 | 3.377* | 8.766** | 5.145* | 36.691*** | 6.546** | 4.086* | 3.622* |
| Soil depth | | 28.641*** | 28.387*** | 2.728 | 35.722*** | 2.313 | 0.050 | 2.905 | 8.314* |
| Elevation × Soil depth | | 1.064 | 0.110 | 0.868 | 0.577 | 0.076 | 0.115 | 0.066 | 0.941 |

SWC, soil water content; SBD, soil bulk density; STN: soil total nitrogen; STP, soil total phosphorus; STK, soil total potassium; ECa, exchange of calcium; EMg, exchangeable magnesium.

**Table A2** Litter C, N, and P contents and their stoichiometric ratios at different elevations.

| Elevation (m asl.) | Litter C | Litter N | Litter P | Litter C:N | Litter C:P | Litter N:P |
| --- | --- | --- | --- | --- | --- | --- |
| 200 | 362.98 ± 45.89 AB | 15.12 ± 1.48 A | 1.29 ± 0.12 A | 24.24 ± 4.67 B | 284.39 ± 63.40 B | 11.71 ± 0.88 C |
| 300 | 343.11 ± 14.33 B | 14.19 ± 0.90 A | 0.67 ± 0.11 B | 24.29 ± 2.59 B | 522.36 ± 114.99 B | 21.33 ± 2.37 B |
| 400 | 359.28 ± 46.66 AB | 14.09 ± 1.59 A | 0.64 ± 0.08 B | 25.99 ± 6.58 B | 568.60 ± 134.56 B | 21.94 ± 1.34 B |
| 500 | 411.06 ± 17.74 A | 10.92 ± 1.96 B | 0.26 ± 0.07 C | 38.36 ± 6.31 A | 1637.66 ± 446.18 A | 42.21 ± 4.34 A |

Litter C, litter organic carbon; Litter N, litter nitrogen; Litter P, litter phosphorus; Litter C:N, litter organic carbon: litter nitrogen; Litter C:P, litter organic carbon: litter phosphorus; Litter N:P, litter nitrogen: litter phosphorus.
